# Ilizarov bone transport and treatment of critical-sized tibial bone defects: a narrative review

**DOI:** 10.1186/s10195-019-0527-1

**Published:** 2019-04-16

**Authors:** Kemal Aktuglu, Kubilay Erol, Arman Vahabi

**Affiliations:** Department of Orthopedics and Traumatology, Ege University, Talatpasa Bulvari, Sezik Apt., No: 61/3 Alsancak, Izmir, Turkey

**Keywords:** Bone defect, Critical size, Ilizarov, Bone transport, Distraction osteogenesis

## Abstract

**Background:**

Critical-sized bone defects of the tibia are complex injuries associated with significant problems that are difficult to treat, and they are associated with a significant burden of disease in clinical practice; however, the treatment of these cases has still been a challenge for orthopedic surgeons. The aim of this review was to evaluate the current available studies reporting on classical Ilizarov methods in the treatment of infected or noninfected critical-sized bone defects of the tibia, and to perform an analysis of treatment period and complications.

**Methods:**

This is a narrative review based on a comprehensive literature search among the studies in Pubmed, Scopus and Web of Science articles. The studies included were written in the English language or translated to English and they were published between 2008 and 2018. They were appraised with narrative data synthesis. The primary outcome measures were the external fixation time (EFT), bone union rate, and bone and functional results. Secondary outcomes were complications including docking site problems and solutions. The heterogeneity of the data in the studies which were taken into consideration allowed a narrative analysis.

**Results:**

Twenty-seven articles with 619 patients were included in this study. These included 6 prospective and 21 retrospective case series. Mean age was 36.1 (range 13–89) years. Of the cases, 88.8% were infected and the remaining 11.2% were noninfected. The external fixation time was 10.75 (range 2.5–23.2) months. The mean bone union rate was 90.2% (range 77–100)%. Radiographic outcome measures were reported in 20 studies. Functional outcome measures were reported in 18 studies. ASAMI (Association for the Study of the Method of Ilizarov) criteria are useful and give reproducible data on patient outcome measurements. Data collected from these studies showed excellent radiological outcomes in 303, good in 143, fair in 31, and poor in 25 patients. Functional outcomes were excellent in 200, good in 167, fair in 58, and poor in 19, where reported. The excellent and good rate in bone results and functional results were 88.8% and 82.6%, respectively. The poor rate in bone results and functional results were 5% and 4.5%. Mean complication rate per patient was 1.22 (range 3–60). The most common complication was pin tract infection (PTI). Its occurrence was 46.6%. Joint stiffness followed PTI with a 25% incidence. The rates of refracture, malunion, infectious recurrence, and amputation, were 4%, 8.4%, 4.58%, and 1%, respectively.

**Conclusions:**

This narrative review shows that the patients with infected or noninfected critical-sized tibial bone defects treated by Ilizarov methods had a low rate of poor bone and functional results. Therefore, Ilizarov methods may be a good choice for the treatment of infected or noninfected tibial bone defects. The small number of cases in some studies, the absence of homogenity between studies and the fact that most data available are derived from retrospective studies are some of the difficulties encountered in the evaluation of evidence.

**Level of evidence:**

V.

## Introduction

Infected or noninfected critical-sized tibial bone defects (CSBD) are common in clinical practice [1–3]; however, the treatment of these conditions has still been a challenge for orthopedic surgeons with even greater challenges in the presence of infection or associated soft tissue defects [4, 5]. There is not one standard definition of a critical-sized defect. CSBD are defined as those that will not heal spontaneously within a patient’s lifetime [1, 2]. Several methods have been applied successfully in the treatment of infected or noninfected tibial CSBD, including bone grafting, free tissue transfer and antibiotic cement, but these treatments have obvious limitations, such as donor site morbidity, stress fracture, and restriction of the size of bone defects [2]. The primary contemporary means of reconstructing CSBD are the induced membranes technique, pioneered by Masquelet, and distraction osteogenesis (DO), introduced by Ilizarov. Both of these methods have modifications widely used today in large bone defects. In the last decade, the induced membrane technique, also known as the Masquelet technique, the classic Ilizarov method, and the modification of the Ilizarov bone transport method have gained popularity.

The Ilizarov method for the treatment of complex tibial pathology associated with CSBD generally involves bifocal or trifocal bone transport. Bone transport is characterized by the gradual translocation of a segment of bone from a healthy area into a region of bone loss [4, 5]. Up to now, there have been numerous reports on the treatment of tibial bone defects by Ilizarov methods, and they have gradually become main treatments for infected tibial bone defects. Although bone defects treated by Ilizarov methods reached a satisfactory outcome in most studies, there were still some relatively unsatisfactory results in several studies [5, 6]. In addition, a relatively high rate of complication in Ilizarov methods has been reported in some clinical research [7, 8].

The systematic reviews and meta-analyses done before in this field were mainly based on the evaluations of smaller series. The results obtained from this study should be evaluated along with the results of previous studies so that the place of classic Ilizarov treatment in infected or noninfected tibial bone defects can be understood better. In this narrative review, studies from the last 10 years, based on the traditional Ilizarov method, were taken into consideration. Although the number of Ilizarov-like cases is very high, due to many modifications made to the method we do not have homogenous studies to work on. In some studies, the traditional circular frame with a standard Ilizarov procedure is compared to hybrid and other types of fixators. Only classic Ilizarov related information was taken into consideration from these studies [4–6].

## Historical aspect of distraction osteogenesis

The Ilizarov method, which is used in the treatment of complicated fractures of long bones, was first introduced in 1950 by Gavril Abramovich Ilizarov, in the Soviet Union. This revolutionary method for treating fractures, nonunions, deformities and other bone defects involved the use of a circular external fixator [7, 8]. In 1965, Russian high jump gold medalist Valery Brummell had a motorcycle accident that resulted in an injury on his right tibia. He had 29 operations and his leg remained in a cast for 3 years. The amputation of the leg was discussed, and his treatment was undertaken by Gavril Ilizarov. After his treatment, Brummell’s leg was saved and he was able to jump again. Bone transport was the key to success in this treatment. Starting in the early 1950s, he worked in a village in Siberia, Kurgan, unknown to the rest of the world. After his success in treating Brummell, Ilizarov became known in Russia. Then, in 1982, he successfully treated a famous Italian explorer Carlo Mauri for a resistant nonunion of his tibia and it was only then that his principles were made known to the Western world [7–12].

## Materials and methods

**Fig. 1:**
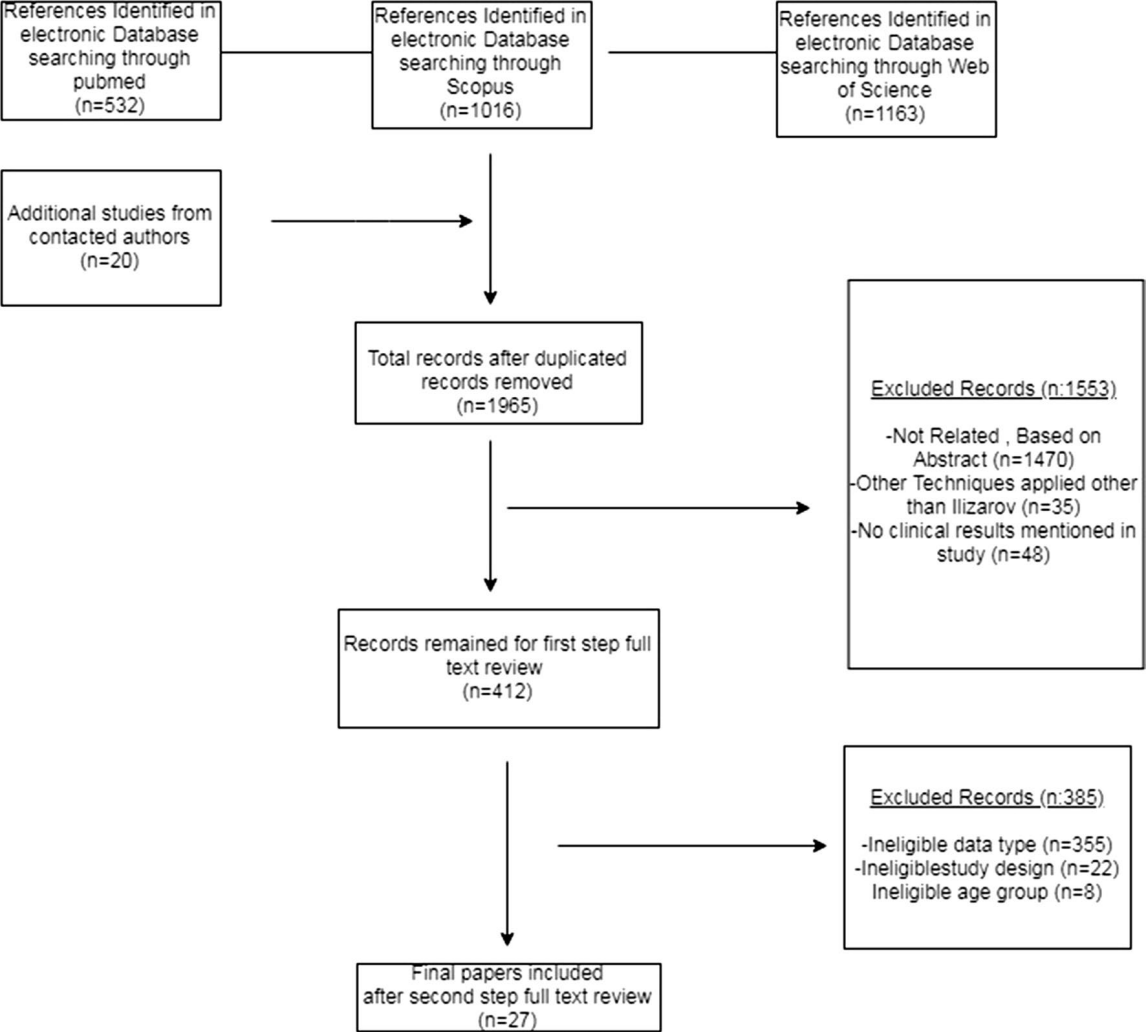
Flow chart of the literature review

PubMed, Scopus and Web of Science databases were searched to identify articles published between January 2008 and December 2018 pertinent to the methods and outcomes of surgical treatment of CSBD by classic Ilizarov method. The standardized treatment included bacterial eradication by segmental resection, bone transport using an Ilizarov circular external fixator, and docking maneuver. The keywords used to identify relevant articles were ‘bone defect’ or ‘critical size(d)’, ‘bone transport’, ‘distraction osteogenesis’ and/or ‘large’, ‘tibia(l)’ and/or ‘Ilizarov’ (Fig. 1). With these keywords, 2711 articles were identified. In addition to those 2711 studies, 20 more studies from contacted authors were added. After removing duplicate studies, 1965 remained. Of these 1965, 1553 were excluded from the analysis because they failed to meet the surgical treatment criteria or to report postoperative outcomes, ineligible data type, ineligible study design, ineligible population or were case reports or series with fewer than 5 patients. There were 412 studies left for the first step of full text screening; 385 more studies were excluded in the full text review step, which failed to meet the above mentioned criteria.

If the information about the Ilizarov method was not clear enough, or insufficient, they were not considered as part of this report. The inclusion criteria for this study were as follows: the target population was patients with infected or noninfected CSBD; intervention methods were Ilizarov methods, including bone transport, the outcomes included were bone union; bone results and functional results were evaluated by ASAMI, complications, EFT and EFI. Eligible studies included two above-mentioned outcomes, at least. Articles, excluding small case reports, and reviews written completely in English were considered. Duplicate or multiple publications of the same study were excluded. Studies involving pediatric series, non-traumatic bone defects, unilateral frame external fixator applications, lengthening over an intramedullary nail, free tissue transfer with distraction osteogenesis, animal models, basic research, abstracts, soft tissue repairs, and reports evaluating femoral and tibial bone defects together were excluded, and when it was impossible to extract or calculate the information about outcomes and/or surgical treatment of CSBD from the studies, these were eliminated. Patients were excluded from the study if they had neurological disorders affecting gait, or reported any systemic bone disease.

As a result, 27 studies and a total of 619 patients were eligible to be included in our analysis [13–39]. They fulfill the inclusion criteria, and data from these studies were reviewed. In this article, a study of cases where the traditional Ilizarov technique was used to treat infected or noninfected critical-sized tibial bone defects, published between 2008 and 2018 are presented. All relevant data that met the eligibility criteria were independently and separately extracted by three authors. Discrepancies were resolved by discussion with each other.

## Results

Twenty-seven major studies published between 2008 and 2018 were identified in the literature that studied application of the Ilizarov technique on patients with large tibial bone defects. Twenty-one retrospective studies formed the preponderance of the body of research. The remainder included 6 prospective cohorts [13, 18, 19, 22, 25, 33]. Out of the 27 studies, the treatment periods for the cases were not reported in 2 studies [20, 34]; the mean for the remaining 25 studies was 5 (range 1–19) years. The average number of cases in each study was 24 (range 7–86).

In 6 of the 27 studies, infected and noninfected CSBD were mixed [19, 25, 29–32, 39]. Eighteen studies included only infected tibial CSBD. When all 619 cases were taken into consideration, 535 of them were reported as infected bone defect, and 57 were reported as noninfected bone defect; the remaining 10 cases were acute trauma [21]. In one study, 17 cases were not reported in these terms [18]. The interventions mainly included three parts: radical debridement, antibiotic treatment, and Ilizarov methods, which included only the bone transport technique. The Ilizarov method for the treatment of CSBD of the tibia generally involves bifocal or trifocal osteosynthesis. A bone defect longer than 6 cm was an indicator of tri-focal transport [17]. There were 507 (507/619) patients that received bifocal bone transport technique, 43 patients received trifocal bone transport technique, and for the remaining patients, there was no clear data. Further details are listed in Tables 1 and 2.

**Table 1:** Descriptive characteristics of component studies

Author/s	Published year	Study design	Treatment period	Number of patients	Mean age (range)	Male/female ratio	Follow-up rate (%)	Mean follow-up time (range) (months)
Madhusudhan et al.	2008	PC	3 years	22	37.2 (20–52)	18/4	100	13 (6–20)
Pirwani et al.	2008	RS	2004–2006	16	32 (20–60)	16/0	100	16 (12–27)
Bumbasirevic et al.	2010	RS	1991–1996	30	30.4 (20–48)	29/1	100	99 (62–122)
Megas et al.	2010	RS	1998–2005	9	39.7 (21–75)	7/2	100	26.6 (16–42)
Lin et al.	2012	RS	1997–2012	16	36 (18–70)	nr	93.7	nr
Babar et al.	2013	PC	2009–2011	17	32.7 (18–52)	15/2	100	nr
Feng et al.	2013	RS	nr	21	34.6 (19–49)	15/6	90.4	31 (12–72)
Krappinger et al.	2013	PC	2004–2009	15	32 (16–61)	11/4	100	17.3 (nr)
Selim	2013	RC	2010–2011	10	30 (22–40)	10/0	100	28.8 (24–36)
Shadid et al.	2013	RC	2009–2010	12	43.4 (28–89)	10/2	100	14.25 (nr)
Spiegl et al.	2013	PC	2006–2009	25	46 (20–60)	22/3	100	29.4 (25–38)
Xu et al.	2013	RS	2003–2011	30	34.1 (19–49)	21/9	100	29 (12–72)
Yin et al.	2014	RS	2004–2011	66	37.06 (nr)	62/4	90	25.91 (18–46)
Morsy	2014	PC	2010–2013	12	36.5 (24–48)	10/2	100	9.2 (6–20)
Marais et al.	2014	RC	2009–2013	7	29 (28–44)	nr	85,7	28 (nr)
Azzam et al.	2015	RS	2011–2013	30	32 (18–52)	30/0	100	18 (10–32)
Bernstein et al.	2015	RC	2006–2012	30	43 (25–56)	24/6	100	31 (nr)
Khan et al.	2015	RS	2005–2010	24	38 (13–74)	21/3	100	11 (8–46)
Peng et al.	2015	RS	2008–2011	58	29 (18–51)	38/20	100	31 (24–63)
Wani et al.	2015	RS	2010–2012	26	39 (20–65)	22/4	100	nr
Aboumira et al.	2016	RC	1999–2001	25	44.5 (21–75)	19/6	100	53 (25–74)
Aktuglu et al.	2016	RS	1995–2013	24	35.04 (8–69)	21/3	100	74.08 (39–122)
Fürmetz et al.	2016	RS	2000–2010	8	39 (27–54)	7/1	100	46 (nr)
Rohilla et al.	2016	PC	2008–2013	35	36.1 (12–60)	30/5	97.5	25.4 (6–48)
Tetsworth et al.	2017	RC	nr	21	38.2 (nr)	18/3	100	25.5 (12–84)
Yilihamu et al.	2017	RS	1996–2015	14	35.9	11/3	100	96 (nr)
Zhang et al.	2018	RS	2010–2015	16	39.1 (16–65)	9/7	100	29.5 (nr)

*nr* not reported, *PC* prospective cohort, *RS* retrospective study, *RC* retrospective cohort

**Table 2:** Interventions and outcomes of included studies

Author/s	Technique	Bone union rate (%)	Bone results (ASAMI, PALEY) (excellent/good/fair/poor)	Functional results (ASAMI, PALEY) (excellent/good/fair/poor)	Complications (per patient)	EFT (months)	EFI (months/cm)
Madhusudhan et al.	RD/AT/ACL (IEF)	100	5/8/5/4	1/4/6/10^a^	2.73 (60/22)	9.3	2.33
Pirwani et al.	RD/AT/BT (IEF)	100	nr	nr	2 (32/16)	16	3
Bumbasirevic et al.	RD/AT/BT (IEF)	96.6	19/10/0/1	13/14/2/1	1.4 (42/30)	9.7	1.48
Megas et al.	RD/AT/CO or ACL	100	5/4/0/0	3/4/2/0	1.89 (17/9)	7.83	1.07
Lin et al.	RD/AT/BT (IEF)	93.7	nr	nr	1 (16/16)	4.5	nr
Babar et al.	RD/AT/BT (IEF)	97	13/2/2/0	10/4/2/1	1 (17/17)	6	nr
Feng et al.	RD/AT/BT (IEF)	100	19/2/0/0	nr	0.4 (8/21)	9.8	1.48
Krappinger et al.	RD/AT/BT (IEF)	80	7/6/2/0	6/7/2/0	3 (45/15)	13.2	nr
Selim	RD/AT/BT (IEF)	80	7/3/0/0	7/3/0/0	0.7 (7/10)	2.5	0.28
Shadid et al.	RD/AT/ACL (IEF)	100	10/2/0/0	6/4/2/0	0.25 (3/12)	nr	nr
Spiegl et al.	RD/AT/BT (IEF)	96	nr	nr	1.36 (34/25)	23.2	1.9
Xu et al.	RD/AT/BT (IEF)	100	28/2/0/0	nr	0.27 (8/30)	10	1.36
Yin et al.	RD/AT/BT (IEF)	100	44/15/5/2	24/26/10/0^b^	1.1 (73/66)	9.4	1.38
Morsy	RD/AT/BT (IEF)	100	8/3/0/1	7/4/1/0	1.58 (19/12)	6.8	1.52
Marais et al.	RD/AT/BT (IEF	100	nr	nr	1.57 (11/7)	17.7	2.7
Azzam et al.	RD/AT/BT (IEF)	100	22/6/1/1	13/9/7/1	1.7 (51/30)	7.5	1.3
Bernstein et al.	RD/AT/BT (IEF)	77	nr	nr	0.77 (17/22)	11.03	2.5
Khan et al.	RD/AT/CO or ACL (IEF)	95.7	6/14/1/2^c^	8/12/2/1^c^	0.5 (12/24)	8	4.2
Peng et al.	RD/AT/BT (IEF)	100	30/23/5/0	28/18/12/0	0.67 (39/58)	10.6	1.2
Wani et al.	RD/AT/BT (IEF)	100	13/9/4/0	9/11/5/1	2 (52/26)	14.07	1.6
Aboumira et al.	RD/AT/BT (IEF)	89	11/8/3/3	11/9/2/3	0.72 (18/25)	11.8	2.1
Aktuglu et al.	RD/AT/BT (IEF)	95.8	12/8/2/2	14/9/1/0	1 (24/24)	11.52	1.73
Fürmetz et al.	RD/AT/BT (IEF)	100	nr	nr	nr	9.52	1.47
Rohilla et al.	RD/AT/BT (IEF)	94	19/13/0/3	14/19/1/1	1.2 (42/35)	11.9	1.8
Tetsworth et al.	RD/AT/BT or ACL (IEF)	100	15/5/1/0	14/6/1/0	3.1 (nr)	12.5	1.8
Yilihamu et al.	RD/AT/BT (IEF)	nr	nr	nr	1.46 (21/14)	9.8	1.51
Zhang et al.	RD/AT/BT (IEF)	100	10/0/0/6	12/4/0/0	1.18 (19/16)	12	1.1

*nr* not reported, *ACL* acute compression and lengthening, *ASAMI, PALEY* Association for the Study of the Method of Ilizarov, *AT* antibiotics treatment, *BT* bone transport, *CO* compression osteosynthesis, *EFI* external fixation index, *EFT* external fixation time, *IEF* Ilizarov external fixator, *RD* radical debridement

^a^1 patient lost in follow-up

^b^6 patients lost in follow-up

^c^1 patient died from liver disease

The mean age at the time of injury was 36.1 years (range 13–89), and most patients were men (496 men, 100 women and for 23 cases the gender was not specified) [13–39] (Table 1). The mean delay from injury to Ilizarov bone transport treatment was 11.6 months (range 1–62); this value was not reported in 11 studies. The patients had an average of 3.44 (range 1–35) previous surgical procedures before receiving treatment by the Ilizarov method; 11 studies did not report this information [13–39]. The mean delay from injury to Ilizarov treatment and the mean previous operative procedures were both not reported in the same 9 studies (Table 3).

**Table 3:** Details of applied treatments

Author/s	Mean delay from injury to Ilizarov treatment (range) months	Mean previous operative procedures (range)	Etiology n (%)		
			Acute trauma	Aseptic lesion	Infected lesion
Madhusudhan et al.	7.8 (nr)	3 (2–5)	0 (0)	0 (0)	22 (100)
Pirwani et al.	nr	nr	0 (0)	0 (0)	16 (100)
Bumbasirevic et al.	8.6 (16–24)	1.3 (1–3)	0 (0)	0 (0)	30 (100)
Megas et al.	7.8 (4–14)	4.8 (3–6)	0 (0)	0 (0)	9 (100)
Lin et al.	nr	nr	0 (0)	0 (0)	16 (100)
Babar et al.	nr	nr	nr	nr	nr
Feng et al.	8.6 (6–24)	6 (3–14)	0 (0)	0 (0)	21 (100)
Krappinger et al.	13 (10–41)	10.1 (2–35)	0 (0)	6 (40)	9 (60)
Selim	nr	nr	10 (100)	0 (0)	0 (0)
Shadid et al.	21 (1–62)	1.08 (1–2)	0 (0)	0 (0)	12 (100)
Spiegl et al.	9.5 (1–22.6)	1 (nr)	0 (0)	0 (0)	25 (100)
Xu et al.	8.8 (6–24)	6 (3–14)	0 (0)	0 (0)	30 (100)
Yin et al.	22.8 (4–10)	2.4 (1–8)	0 (0)	0 (0)	66 (100)
Morsy	18.2 (7–26)	nr	0 (0)	7 (58)	5 (42)
Marais et al.	3 (nr)	nr	0 (0)	0 (0)	7 (100)
Azzam et al.	nr	nr	0 (0)	8 (26.7)	22 (73.3)
Bernstein et al.	nr	nr	0 (0)	14 (47)	16 (53)
Khan et al.	11.9 (1–36)	2 (nr)	0 (0)	0 (0)	24 (100)
Peng et al.	7.1 (1.4–11.6)	6.3 (3–10)	0 (0)	0 (0)	58 (100)
Wani et al.	8.2 (4.6–28)	2.5 (1–5)	0 (0)	0 (0)	26 (100)
Aboumira et al.	nr	nr	0 (0)	7 (28)	18 (72)
Aktuglu et al.	nr	3.64 (0–11)	0 (0)	8 (33.3)	16 (66.6)
Fürmetz et al.	nr	nr	0 (0)	7 (87.5)	1 (12.5)
Rohilla et al.	5.8 (0.9–22.8)	1.22 (nr)	0 (0)	0 (0)	35 (100)
Tetsworth et al.	nr	4.5 (nr)	0 (0)	0 (0)	21 (100)
Yilihamu et al.	nr	nr	0 (0)	0 (0)	14 (100)
Zhang et al.	16.9 (3–45)	4.25 (nr)	0 (0)	0 (0)	16 (100)

*nr* not reported

The mean bone defect was 6.58 cm (range 1.6–20) according to 25 studies; there was no clear information from 2 studies [35, 38] (Table 4). Minimum CSBD was over 3.0 cm in 24 of the studies. Minimum bone defect sizes were 1.6 cm in Bernstein et al., 2.0 cm in Megas et al. and Khan et al., and 2.5 cm in Wani et al. studies.

**Table 4:** Further details of applied treatment

Author/s	Bone defect		Mean latency period (range) (days)	Type of Ilizarov treatment (n, %)		
	Incidence (%)	Mean size (range) (cm)		Bifocal	Trifocal	Bifocal or trifocal (nr)
Madhusudhan et al.	100	4 (2–11)	nr (5–7)	22 (100)	0 (0)	0 (0)
Pirwani et al.	100	4.5 (2–8)	nr	16 (100)	0 (0)	0 (0)
Bumbasirevic et al.	100	6.9 (4–11)	7	30 (100)	0 (0)	0 (0)
Megas et al.	100	5 (2–12)	nr (3–5)	6 (66.6)	0 (0)	3 (33.3)
Lin et al.	100	8 (4–12)	7	8 (50)	8 (50)	0 (0)
Babar et al.	100	5.8 (nr)	7	17 (100)	0 (0)	0 (0)
Feng et al.	100	6.6 (3–12)	7	21 (100)	0 (0)	0 (0)
Krappinger et al.	100	6.6 (3–14.7)	nr (10–14)	15 (100)	0 (0)	0 (0)
Selim	100	9 (6–12)	7	0 (0)	10 (100)	0 (0)
Shadid et al.	nr	nr	nr	0 (0)	0 (0)	12 (100)
Spiegl et al.	100	5.3 (3–13)	7	25 (100)	0 (0)	0 (0)
Xu et al.	100	6.4 (3–12)	7	0 (0)	0 (0)	30 (100)
Yin et al.	100	6.27 (3–13)	nr (7–10)	66 (100)	0 (0)	0 (0)
Morsy	100	4.6 (4–7)	nr (7–10)	12 (100)	0 (0)	0 (0)
Marais et al.	100	7 (5–8)	7	7 (100)	0 (0)	0 (0)
Azzam et al.	100	7.4 (3.–12)	7	30 (100)	0 (0)	0 (0)
Bernstein et al.	100	5.9 (1.6–13)	nr	30 (100)	0 (0)	0 (0)
Khan et al.	100	3.2 (2–5)	nr	8 (33.3)	0 (0)	16 (66.6)
Peng et al.	100	9.2 (6–15)	10 (nr)	58 (100)	0 (0)	0 (0)
Wani et al.	100	5.1 (3–8)	7	18 (75)	0 (0)	8 (25)
Aboumira et al.	100	6.5 (3–17)	nr (12–14)	16 (64)	9 (36)	0 (0)
Aktuglu et al.	100	7.01 (5–18)	nr (5–7)	24 (100)	0 (0)	0 (0)
Fürmetz et al.	100	9 (3.1–13.4)	7	8 (100)	0 (0)	0 (0)
Rohilla et al.	100	7.27 (6–12)	7	35 (100)	0 (0)	0 (0)
Tetsworth et al.	100	7 (3–10)	nr	21 (100)	0 (0)	0 (0)
Yilihamu et al.	100	nr (> 3)	10	14 (100)	0 (0)	0 (0)
Zhang et al.	100	10.9 (6–20)	nr (7–10)	0 (0)	16 (100)	0 (0)

*nr* not reported

Mean follow-up rate was 100% in 22 studies but was 93.7% for Lin et al., 90.4% for Feng et al., 90% for Yin et al., 85.7% for Marais et al. and 97.5% for Rohilla et al. [17, 20, 24, 26, 33]. Overall mean follow-up rate was 98.38% (range 87.5–100). When specified in the study, mean follow-up duration was 34.05 months (range 6–122), which was long enough to evaluate clinical and radiological outcomes in most cases [13–39]. Further details are listed in Table 1.

The main disadvantage of the Ilizarov method is the lengthy EFT. The mean EFT was 10.7 months (range 2.5–23.2) for the patients in this review [13–39]. There was no clear information in 1 out of 27 studies about the mean EFT [38]. The mean EFI was 1.74 months/cm (range 0.28–4.2) in the patients [13–39]. The mean EFI was not reported in 4 studies [17–19, 38]. In some studies, EFI (mean 1.36 months/cm, when specified) was used to evaluate bone healing progress, in some studies a healing index (mean 45.57 days/cm, when specified) was used instead [19, 21, 22, 28, 35–37]. Further details are listed in Table 2.

The mean bone union rate was 100% for 15 studies and not reported in one study. It was 95.8% for the remaining 11 studies [13–39]; in 26 studies mean bone union was 90.2% (range 77–100). In these studies, 242 cases had bone union without any problems at the docking site with the external fixator, without any bone grafting. In 193 cases bone grafting at the docking site was routinely performed in all patients immediately after finishing transport. In only 15 cases was early freshening of fracture ends and removal of interposing soft tissue at the docking site performed to achieve union. In the remaining 202 cases, late grafting because of nonunion, plating and intramedullary nailing were performed, and there is not enough information to determine what has been done. Consolidation at the docking site and at the regenerated bone occurred in 49 (89%) of 55 cases after the first procedure [29]. Bone grafting at the docking site is frequently necessary after bone transport is complete. Also, delayed union of the docking side required İliac crest bone graft. Bone grafting as a routine treatment was recommended in 3 included studies [17, 19, 31].

In this review, when the mean complication per patient was evaluated, it was found that 1 out of 27 did not have sufficient information [32] (Table 2). Two of the remaining 26 studies classified complications according to the Paley system. Spiegl et al. found that the average complication rate per patient consists of 0.88 minor and 0.52 major complications [22]. Tetsworth et al. on the other hand, found 1.2 minor and 1.0 major complications [34]. When the remaining studies were reviewed, the mean complications per patient were 1.22 (range 3–60) [13–39].

Spiegl performed bone transport through induced membrane for post-infective tibial defects in excess of 4 cm. His conclusion was that this procedure was futile. Also, there are studies in favor of and opposed to antibiotic cement spacer application [26, 37]. There is not enough clear information on this. Radical debridement is the most important step for eradicating infection of bone and soft tissue.

The most common complications with Ilizarov fixators were PTI and joint stiffness; details are in Table 5. Superficial PTI and/or joint stiffness were seen in 410 cases out of 619. When the studies were evaluated according to the frequency of PTI, 3 studies out of 27 had 100% PTI [1, 2, 25] and 2 studies had no information about PTI [17, 32]. Mean PTI frequency in the studies was 9 (range 1–40). In two studies dated 2008, it was reported that PTI was seen in all cases [13, 14]. The percentage of this frequency was 46.6% (range 10–100). Out of 595 patients who were evaluated in the studies, with regard to information on PTI, 299 had PTI. PTI was especially reported as the most commonly seen complication [19, 22, 24, 25, 28, 30, 33, 37].

**Table 5:** Complications of Ilizarov methods

Author/s	Number of patients	Pin tract infection (n)	Wire breakage (n)	Refracture (n)	Joint stiffness (n)	Amputations (n)	Equinus deformity (n)	Docking site problems (n)	Malunion (angular deformity) (n)	Persistent infection (n)	Regeneration problems (n)	Others
Madhusudhan et al.	22	22	7	nr	22	0	22	nr	nr	6	1	2 knee septic arthritis 22 limb oedema
Pirwani et al.	16	16	0	nr	0	0	0	2	0	2	1	nr
Bumbasirevic et al.	30	14	0	0	16	0	19	1	4	nr	nr	1 peroneal neuritis 2 chronic pain 4 limb oedema
Megas et al.	9	8	0	nr	5	0	nr	0	2	0	0	nr
Lin et al.	16	nr	nr	nr	nr	nr	nr	0	0	1	nr	nr
Babar et al.	17	8	3	nr	nr	nr	nr	1	nr	0	1	nr
Feng et al.	21	3	nr	nr	0	0	0	nr	2	0	nr	nr
Krappinger et al.	15	9	nr	nr	5	0	0	3	8	2	2	nr
Selim	10	1	0	1	3	0	2	2	0	0	0	nr
Shadid et al.	12	2	1	0	0	0	0	nr	0	nr	0	nr
Spiegl et al.	25	9	0	nr	0	1	1	5	5	7	0	nr
Xu et al.	30	3	1	nr	0	0	0	nr	2	0	0	1 early mineralization
Yin et al.	66	40	4	2	nr	0	nr	6	0	0	nr	nr
Morsy	12	12	0	nr	1	0	1	1	1	1	3	nr
Marais et al.	7	2	0	1	1	1	1	2	0	0	0	2 flap dehiscence
Azzam et al.	30	nr	1	1	14	0	0	1	4	1	1	1 residual length discrepancy 1 skin invagination
Bernstein et al.	30	5	0	3	0	0	3	1	3	nr	0	1 knee septic arthritis 2 skin entrapment
Khan et al.	24	5	0	nr	0	1	1	0	1	1	0	2 skin entrapment
Peng et al.	58	18	1	0	5	0	1	0	0	0	0	0
Wani et al.	26	23	0	nr	8	0	0	3	9	0	1	2 residual length discrepancy
Aboumira et al.	25	14	3	2	0	0	1	2	1	2	4	nr
Aktuglu et al.	24	17	0	nr	7	0	3	5	0	0	0	6 residual length discrepancy
Fürmetz et al.	8	nr	nr	nr	nr	2	nr	nr	nr	nr	nr	nr
Rohilla et al.	35	25	2	1	25	1	0	0	8	0	0	nr
Tetsworth et al.	21	8	1	1	5	0	1	7	0	nr	9	nr
Yilihamu et al.	14	5	0	nr	8	0	2	4	0	0	0	2 knee dislocation 1 ankle dislocation
Zhang et al.	16	13	3	1	3	0	0	3	0	nr	1	nr

*nr* not reported

For 128 patients in 23 studies, ankle or joint stiffness was reported. But there was no clear distinction reported and the mean percentage was 25% (range 0–100). In some studies, equinus deformity was noted under a separate column. In the 4 studies where there was no mention of joint stiffness, equinus deformity was reported [22, 27, 29, 39]. Of the 27 studies, 22 reported equinus deformity. Mean equinus deformity was 12% (range 0–100) when the cases were evaluated according to ankle and knee joint problems in 23 studies; at the end of the treatment, 95 cases had ankle contracture and/or equinus deformity, 46 cases had knee joint stiffness and 20 cases had both knee and ankle contracture on the same side. Some ankle stiffness or equinus deformities were related to treatment, while some were due to a previous injury or treatment: the causes were not clear-cut. Spiegl et al. reported 5 patients (20%) who required upper ankle arthrodesis as a sequel of posttraumatic or infectious arthritis, but they were not directly related to Ilizarov procedures [22]. Megas et al. also observed stiffness of the ankle joint in 55% of patients and reported it as a common and severe residual problem after such surgical intervention [16]. Ankle problems are the most important source of residual disability after successful use of the Ilizarov device for the treatment of tibial bone defects. Though knee stiffness was largely overcome with physiotherapy, foot and ankle stiffness persisted and worsened despite bony union. This accounts for the poor outcome in the results. Only Tetsworth et al. reported 3 transient ankle stiffness and 2 transient knee flexion contractures [34]. Poor results are not correlated with ankle and knee joint problems and this shows that the majority of these problems are transient.

When the studies were evaluated according to the healing problems seen at the docking site, there was no information in 4 studies [20, 23, 34, 38]. In 3 studies, fracture healing was achieved without bone grafting at the docking site [13, 16, 30]. In the remaining 20 studies, ABG grafting was performed in 100% of cases for 6 studies. There were 151 cases evaluated in these 6 studies. In 23 studies there were a total of 525 cases, with grafts used in 211 of them, the majority of the grafts being ABG grafts. A total of 36.38% of the cases evaluated in the studies had bone grafting at the docking site. The mean percentage of patients who had docking site grafting was 40.1% (range 3.3–100). Docking site healing problems are summarized in Table 6.

**Table 6:** Details of bone healing problems of docking site

Author/s	Ilizarov cases (n)	Bone grafting n (%)	Graft type	Approach
Madhusudhan et al.	22	0	nr	Ilizarov
Pirwani et al.	16	2 (12.5)	nr	Ilizarov
Bumbasirevic et al.	30	1 (3.3)	ABG	Ilizarov
Megas et al.	9	0	0	Ilizarov
Lin et al.	16	16 (100)	ABG	IMN
Babar et al.	17	4 (23.5)	ABG	Ilizarov (accordion)
Feng et al.	21	nr	nr	Ilizarov
Krappinger et al.	15	15 (100)	nr	Ilizarov
Selim	10	2 (20)	ABG	Ilizarov
Shadid et al.	12	nr	nr	Ilizarov
Spiegl et al.	25	25 (100)	ABG, BMP	Plate/screw
Xu et al.	30	nr	nr	Ilizarov
Yin et al.	66	6 (9)	ABG	Ilizarov
Morsy	12	4 (25)	ABG	Ilizarov
Marais et al.	7	7 (100)	ABG	Ilizarov
Azzam et al.	30	30 (100)	ABG	Ilizarov
Bernstein et al.	22	nr	nr	Ilizarov
Khan et al.	24	0	0	Ilizarov
Peng et al.	58	58 (100)	ABG	Ilizarov
Wani et al.	26	3 (11.5)	ABG	Ilizarov
Aboumira et al.	25	9 (36)	ABG	Ilizarov
Aktuglu et al.	24	0	0	IMN
Fürmetz et al.	8	7 (75)	ABG	Plate/Ilizarov
Rohilla et al.	35	15 (42.8)	ABG	Ilizarov
Tetsworth et al.	21	nr	nr	Ilizarov
Yilihamu et al.	14	4 (28.5)	ABG	Ilizarov
Zhang et al.	16	3 (18.7)	nr	Ilizarov

*nr* not reported, *ABG* autologous bone graft, *BMP* bone morphogenetic protein, *IMN* intramedullary nail

When the studies were evaluated according to regeneration site, there was no information about this in 5 studies [15, 17, 20, 24, 32]. Generally, in the other studies, there was not enough detailed information about the regeneration site. Refracture at the docking site or the regeneration site, after the circular external fixator was removed (between 1 month and 1 year), was seen in a total of 13 (3.82%) cases [21, 24, 26, 31, 33, 34, 36, 39]. Three of these 13 cases happened after tri-focal bone transport and this requires attention [36]. Amputation is one of the risks of infected bone defects and the Ilizarov method can minimize this potential outcome. In this study 6 (1%) of the cases treated with Ilizarov resulted in amputation [22, 26, 27, 32, 33]. These are considered as “failure” in the results. Complications are summarized in Table 5.

When infected CSBD cases were taken into consideration, there were 18 studies available. In this group the mean bone defect size was not reported in two studies [16, 18], in the remaining 16 studies the mean bone defect size was 6.6 cm (range 1.6–20); the number of the mean previous surgical procedures was 3.62 cm (range 1–35), previous surgical procedures were not reported in 11 studies. The mean EFT was 10 months (range 4.5–23.2) and it was not reported in one study. The mean EFI was 1.48 (range 0.52–4.2) and it was not reported in 3 studies. Mean complication rate per patient was 1.22 (range 0.25–3.1). Bone results were excellent in 242 (62%), good in 109 (27.9%), fair in 21 (5.3%) and poor in 18 (4.6%) cases. These results belong to 13 studies and in 5 studies this was not reported. Functional results were excellent in 146 (44%), good in 128 (38.6%), fair in 44 (13.2%) and poor in 13 (3.9%). Eradication of infection and bone union were achieved in almost every patient. Only Spigel et al. reported recurrent infection in seven cases (28%) [22].

The criteria recommended by ASAMI were adopted to evaluate bone results and functional results in the studies. Sometimes, clinical efficacy was assessed using Paley’s grading system and patient satisfaction at the last follow-up [15, 16, 19–21, 23, 29, 37]. Bone results were evaluated in 20 studies by ASAMI criteria with data of 502 patients. The information on the remaining 7 studies was not clear [14, 17, 20, 22, 26, 32, 35]. The bone results were excellent, good, fair and poor in 303 (60.4%), 143 (28.5%), 34 (6.4%) and 25 (5%) patients respectively. The functional results were obtained from 18 studies with 444 patients. The functional results were excellent, good, fair and poor in 200 (45%), 167 (37.6%), 58 (13%) and 19 (4.3%) respectively. The excellent and good rate in bone results and functional results were 88.9% and 82.3%, respectively. Detailed information on bone and functional outcomes are listed in Table 2.

## Discussion

Bone transport can be done through many devices like ring/circular fixators, monolateral fixators or intramedullary nail systems. Each device has its own advantages and disadvantages. Ilizarov fixators have been in use for many years, but very few studies in the literature have focused on outcomes of ring fixators in infected or noninfected CSBD of tibia treated with distraction osteogenesis.

In the literature, only 3 meta-analyses and systematic reviews analyze the effectiveness, complications, and clinical results of Ilizarov methods in the treatment of long bone defects of the tibia. One of these was made by Papakostidis et al. in 2013 [40]. In this study, 37 reports from between 1989 and 2012 were evaluated. Unfortunately, this study took tibial and femoral bone defects together into consideration. Twenty-three of 37 studies evaluated tibial bone defects in 518 cases. The only significant difference that could be established was a fourfold decrease of the likelihood of fair functional results in the tibia subgroup compared with the femur subgroup [40]. The most important message of this study was that the authors noticed a 3.7 times increase in the odds of refracture when the size of the tibial defect exceeded 8 cm.

The second systematic review of tibia infected nonunion treated by the Ilizarov method was made by Yin et al. [24], by reviewing literature from PubMed, Cochrane Library, EMBASE and other relevant English orthopedic journals between January 1995 and April 2013. The initial literature search identified 225 relevant records, and finally 16 studies and a total of 303 patients were included in the systematic review [24]. The following data were calculated: the mean age was 34.4 years (range 25–44); the mean size of bone defects was 6.01 cm (range 3.5–10.7); the mean follow-up was 44.37 months (range 13–99); the bone union rate was 90.2% (range 77–100%); the good and excellent rate in bone results was 87.5% (range 45–100%); the good and excellent rate in functional results was 76% (range 23–97%); the mean number of complications per patient was 1.47 (range 0.12–3.35); the mean EFT was 9.1 months (range 3.1–13.9); and the mean EFI was 1.46 months/cm (range 0.55–2.33).

The third study made by Yin et al. [41] was a systematic review and meta-analysis of Ilizarov methods in the treatment of infected nonunion of tibia and femur. Unfortunately, this also took infected tibias and femurs together into consideration. A comprehensive literature search was performed using SCI, PubMed, Cochrane Library, and EMBASE between January 1995 and August 2015. A total 590 patients with infected nonunion of tibia and femur treated by Ilizarov methods from 24 studies were included in this review [41].

For patients with infected nonunion, the mean age was 34 years and the mean previous number of surgical procedures was 3.84. The mean bone defect length was 6.54 cm and the mean length of follow-up was 32 months. Bone grafting as a routine treatment was recommended in 1 included study. The average bone union rate was 97% in the studies. The mean number of complications for every patient was 1.22, the mean EFT was 9.41 months, and the mean EFI was 1.64 months/cm. Bone results were evaluated in 16 studies by ASAMI. Functional results were reported in 16 studies. The poor rate for bone results and functional results were 8% and 10%, respectively [41].

The rates of refracture and amputation were both 4% in the review by Yin et al. [41], which is similar to the 5% and 2.9%, respectively, reported by Papakostidis et al. [40]. The rates of malunion, infection recurrence, and knee stiffness were, respectively, 7%, 5% and 12%. Pin-track infection is the most common complication in using Ilizarov methods, and significant statistical heterogeneity was found for the complication. The rate of pin-track infection was 10–100% among included studies in our systematic review. The poor rate in bone results and functional results were 7% and 9%. The rates of bone grafting, knee stiffness, malunion, refracture, infection recurrence, and amputation were 7%, 4%, 6%, 13%, 4% and 13%, respectively [41].

Our study was a review of 10 years of data. Papakostidis et al. studied a period of 23 years. Yin et al. performed two separate studies. The first one spanned 18 years and the second one 20 years [24, 40, 41]. Tibia and femur bone defects were taken together in the study by Papakostidis et al., and tibia and femur nonunions were taken together in the second study by Yin et al. When all four studies, including ours, were evaluated, the mean ages of the patients were 34, 34, 34, and 36, respectively. The ratio of bone union for Yin et al.’s first study, which only included tibias, was 96% and for ours it was 90%. But when we looked at excellent and good bone rates, it was 87.5% for Yin et al.’s review and 88.8% in our review. The mean EFT was 9.19 months in Yin et al.’s first study and 10.75 months in our review. The mean EFI was 1.74 months/cm. The mean bone defect length was 6.01 cm in Yin et al.’s study and 6.58 cm in our review. Complications per patient had its lowest rate in our series, at 1.22%. Also, the rate of amputation was lowest in our review, at 1%. The recurrent infection rate was 4.58% in our study: it was at its lowest. The refracture rate was 4% and it was very close to other studies in our review. In our series, malunion was high, at 8.4%; this value was 5.7% for Papakostidis et al., was not reported in Yin et al.’s first study, and was 7% for their second study. PTI was 50.25% in our study but this value was not reported in the other three studies. Details are in Table 7.

**Table 7:** Comparison of rewiews

	Papakostidis et al.	Yin et al.	Yin et al.	Our study
*Characteristics*				
Study period	1989–2012	1995–2013	1995–2015	2008–2018
Number of evaluated studies	37	16	24	27
Number of patients	898	303	590	619
Long bone involved	Tibia + femur	Tibia	Tibia + femur	Tibia
Study types	1 PC, 1P, 1 R, 34 RC	nr	1 PC, 22 R, 1 RC	6 PC, 15 R, 6 RC
Mean age (range)	34.85	34.44 (25–44)	34.11 (nr)	36.1 (13–89)
Mean follow-up (range) (months)	43.81	44.37 (13–99)	32.49 (nr)	34.05 (6–122)
Mean bone union rate (range) (%)	94.3 (92–96.6)	96.69 (87–100)	97.26 (nr)	90.24 (77–100)
Bone results excellent + good (%)	nr	87.52	88.76	88.8
Functional results excellent + good (%)	nr	76	76.70	82.6
EFT (months)	nr	9.19	9.41	10.75
EFI (months/cm)	nr	1.46	1.64	1.74
Mean previous operative procedures (range)	3.46	nr	3.84	3.44
Infection etiology (%)	60.61	100	97.26	88.8
Mean bone defect (range) (cm)	7.37	6.01 (3.5–10.7)	6.54 (nr)	6.58 (1.6–20)
*Complications*				
Complications (per patient)	nr	1.47	1.23	1.22
Refracture (%)	5	nr	4	4
Amputation (%)	2.9	nr	4	1
Malunion (%)	5.7	nr	7	8.41
Recurrent Infection (%)	nr	nr	5	4.58
Pin tract infection (%)	nr	nr	nr	46.6

*nr* not reported, *PC* prospective cohort, *P* prospective, *RC* retrospective cohort, *R* retrospective

To the best of our knowledge, this is the first narrative review of infected and noninfected critical-sized tibial bone defects treated by Ilizarov methods. We were able to provide a large number of data on characteristics of patients and treatment results through 27 included studies.

In conclusion, our review and the current evidence suggest that Ilizarov methods in the treatment of infected or noninfected CSBD resulted in satisfactory effects in bone results and functional results. Radical debridement is the key step in controlling bone infection. However, our review lacks a direct comparison with any other treatment options, and further randomized controlled trials are needed to draw more valuable conclusions. Some studies have declared bone transport using the Ilizarov method for CSBD is the gold standard [32, 36]. The results obtained from this review support this opinion. The most important problem of the classic Ilizarov method is the long duration of the treatment period. Also, PTI, compliance of the patient and the discomfort of the external fixator are other problems that come with this treatment type. Among the modifications made in order to shorten the healing time at the docking site are grafting of the area, and plate and nail application at the site. In order to avoid circular external fixator problems, monolateral fixator usage and bone transport with intramedullary nailing are being used. Although many modifications have been suggested, one of the biggest series consists of classic/traditional applications.

But there are some difficulties in obtaining results from current studies involving traditional Ilizarov methods. The reason for this may be that the authors, when evaluating the cases, did not use homogenous criteria, and did not document the patients in detail. For example, this is very obvious when one looks at regeneration sites in the studies. In the Tables, it can be seen that many items are marked as “not reported”. If the authors use more homogenous criteria for patients, the reporting will be more accurate. Secondly, when evaluating the Ilizarov bone transport method, some studies took femur and tibia together into consideration, and it was impossible to separate the two. These studies were excluded from the review. The information pertaining to these patients has been lost. Another problem is that when comparing the classic/traditional Ilizarov method with any other method in small series, the information was not clear enough, and that made an accurate evaluation hard. A more accurate evaluation could be obtained through more homogenous series.

## Abbreviations

EFI: external fixation index
EFT: external fixation time
PTI: pin tract infection
CSTBD: critical-sized tibial bone defects
DO: distraction osteogenesis
ABG: autologous bone graft
IM: Ilizarov method
IMN: Intramedullary nail
